# A Case of Acute Epidural Hematoma Caused by Mandibular Fossa Fracture

**DOI:** 10.7759/cureus.75023

**Published:** 2024-12-03

**Authors:** Takeyoshi Honta, Noboru Takahashi, Toshio Kikuchi, Shunsuke Omodaka, Hidenori Endo

**Affiliations:** 1 Department of Neurosurgery, Iwate Prefectural Iwai Hospital, Ichinoseki, JPN; 2 Department of Neurosurgery, Tohoku University Graduate School of Medicine, Sendai, JPN

**Keywords:** accidental head trauma, acute epidural hematoma, mandibular fossa fracture, middle cranial fossa fracture, middle meningeal artery (mma)

## Abstract

Acute epidural hematoma is one of the most serious traumatic conditions in neurosurgery, for which emergency surgery may be indicated. Injury to the middle meningeal artery (MMA) is generally the cause of hemorrhage, often accompanied by convexity fractures resulting from head trauma. However, an epidural hematoma by a contusion of the jaw is very rare.

A 27-year-old male fell after drinking alcohol and was brought to our hospital. The patient’s Glasgow Coma Scale (GCS) score was 6 (E1V1M4), and the right pupil was 5 mm with no response to light. A computed tomography (CT) scan of the head showed an acute epidural hematoma in the right middle skull base to the temporal region and no fracture lines of the vault of the skull. An emergency right frontotemporal craniotomy was performed to remove the hematoma. A postoperative recheck of the preoperative images revealed a right mandibular fossa fracture. The postoperative course was uneventful, with clear consciousness and resolution of motor paralysis the day after surgery. On the 11th postoperative day, the patient could walk independently and was discharged. As there was no injury to the MMA in the convexity area, which is generally the cause of acute epidural hematoma, damage to a branch of the MMA resulting from a mandibular fossa fracture was considered a possible mechanism for developing acute epidural hematoma. A case of mandibular fossa fracture resulting in acute epidural hematoma is extremely rare, and the etiology remains unknown, so we reported here with a review of the literature.

## Introduction

Acute epidural hematoma is a serious traumatic disease that is common in the age group of 20-30 years, accounts for 2.7-4% of traumatic brain injuries, and can be fatal [1]. It is generally caused by injury to the middle meningeal artery (MMA) in the convexity. However, hemorrhage from the venous system, mixed arterial and venous hemorrhage, and without fractures due to contrecoup injuries have been reported [2-4]. The present case of acute epidural hematoma resulted from a mandibular fossa fracture. It is extremely rare, with only seven cases, including our case [5]. Thus, we report this case with a review of the literature.

## Case presentation

**Figure 1:**
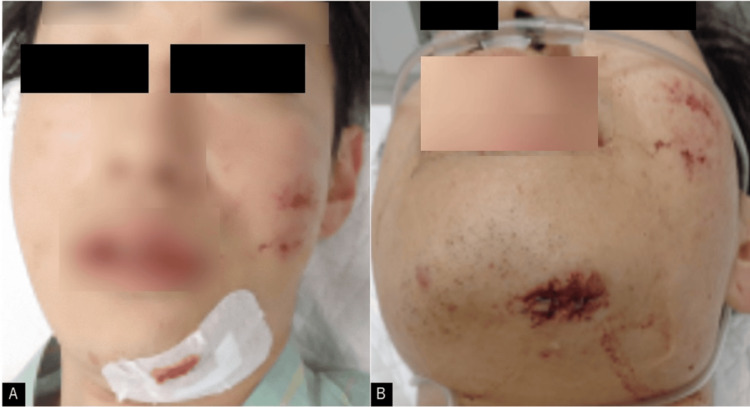
Images taken at the time of admission (A, B) A laceration of the left mandible and a wound on the left face are seen.

A 27-year-old man experienced a fall after alcohol consumption. A few hours later, a passerby found him lying on the ground and called for emergency medical assistance. He was transported to our hospital. On visiting our hospital, the patient’s Glasgow Coma Scale (GCS) score was 6 (E1V1M4). The right pupil was 5 mm in diameter with no response to light, and the left side of the body was completely paralyzed. There was a laceration on the left jaw and a slight abrasion of the face, but no contusion was observed on the head (Figure 1). 

**Figure 2:**
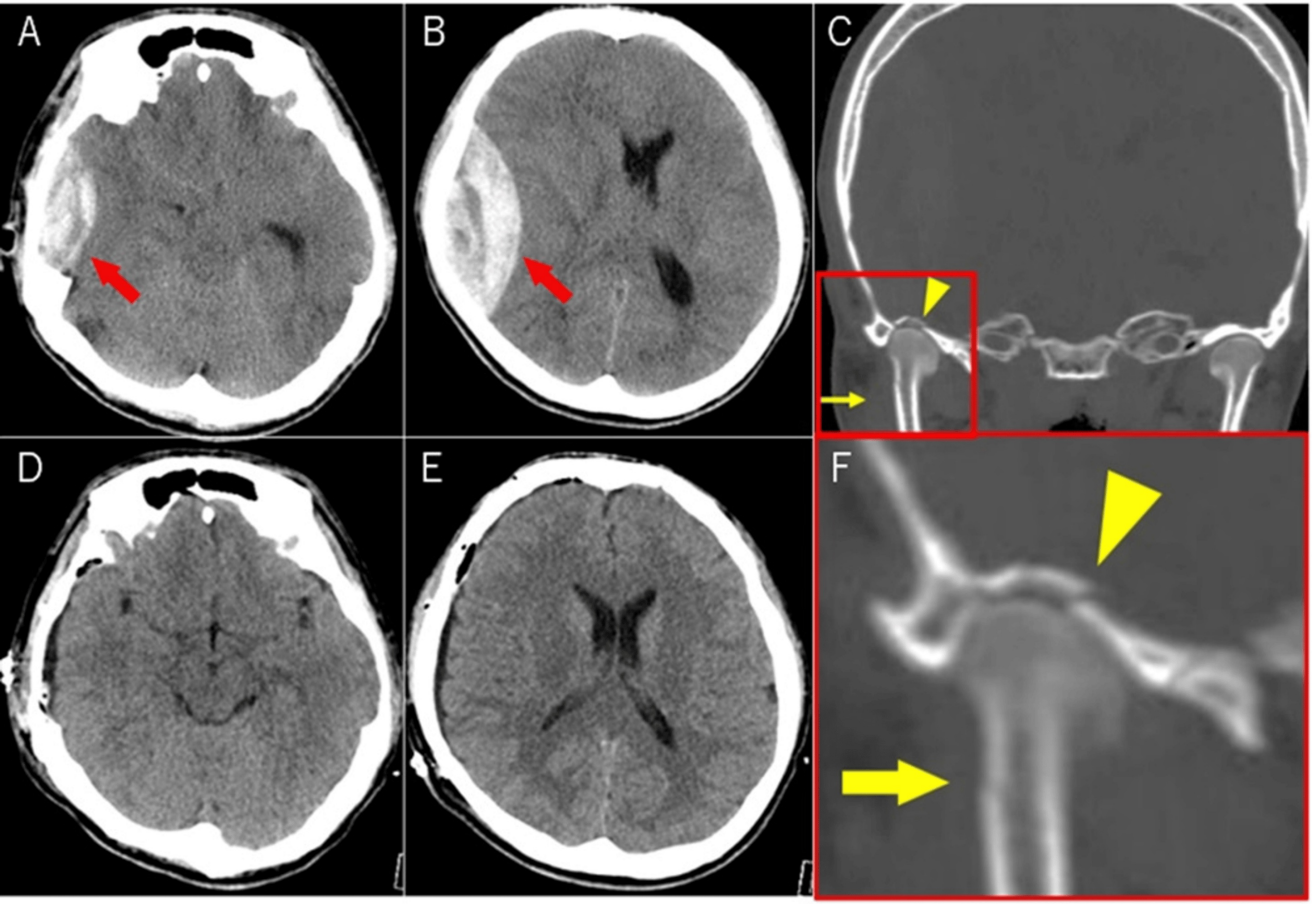
Computed tomography of the head at the time of presentation and postoperatively (A, B) The red arrow indicates acute epidural hematoma from the right middle cranial fossa to the temporal convexity area. (C) shows fracture lines in the right mandibular process (yellow arrow) and mandibular fossa (yellow arrowhead). (D, E) postoperative images. (F) is the magnified image of the fracture site. No fracture line is seen in the convexity area. The operation removed almost all hematoma.

Non-contrast computed tomography (CT) head revealed a right acute epidural hematoma with midline shift and herniation, but no fracture lines of the vault of the skull (Figure 2). This hematoma caused the left-side paralysis of the patient. 

**Figure 3:**
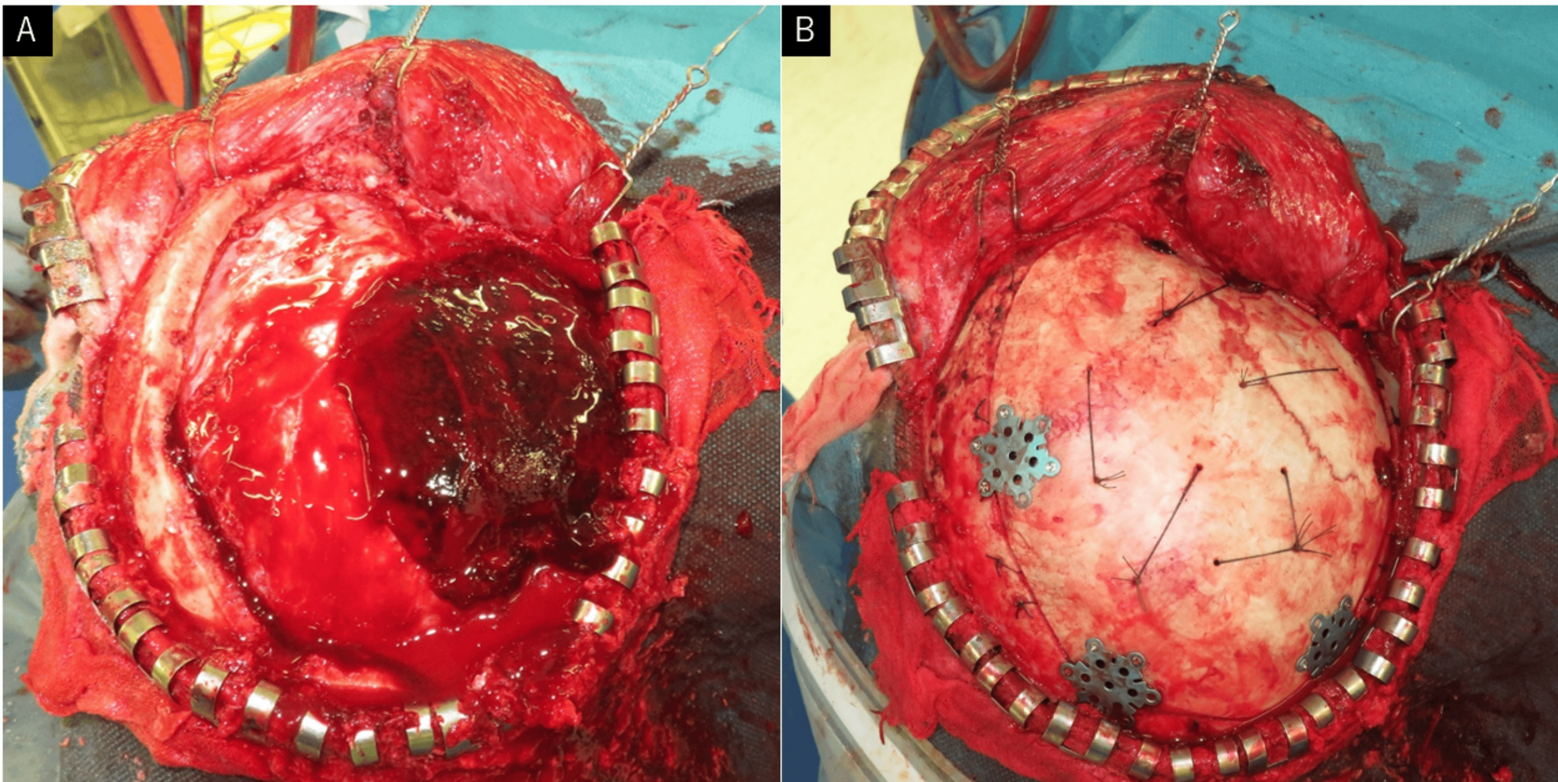
Intraoperative photographs (A) An open craniotomy was performed via the right frontotemporal craniotomy to remove the hematoma. (B) No fracture line was observed intraoperatively in the convexity area.

An emergency right frontotemporal craniotomy was performed to remove the hematoma. Intraoperative findings did not show damage to the MMA in the convexity area (Figure 3). Removal of the hematoma in the middle skull base revealed fracture fragments and hemorrhage in the vicinity of the fracture. Bipolar ablation of the dura and the application of bone wax and compression using oxidized regenerated cellulose were necessary to achieve hemostasis.

**Figure 4:**
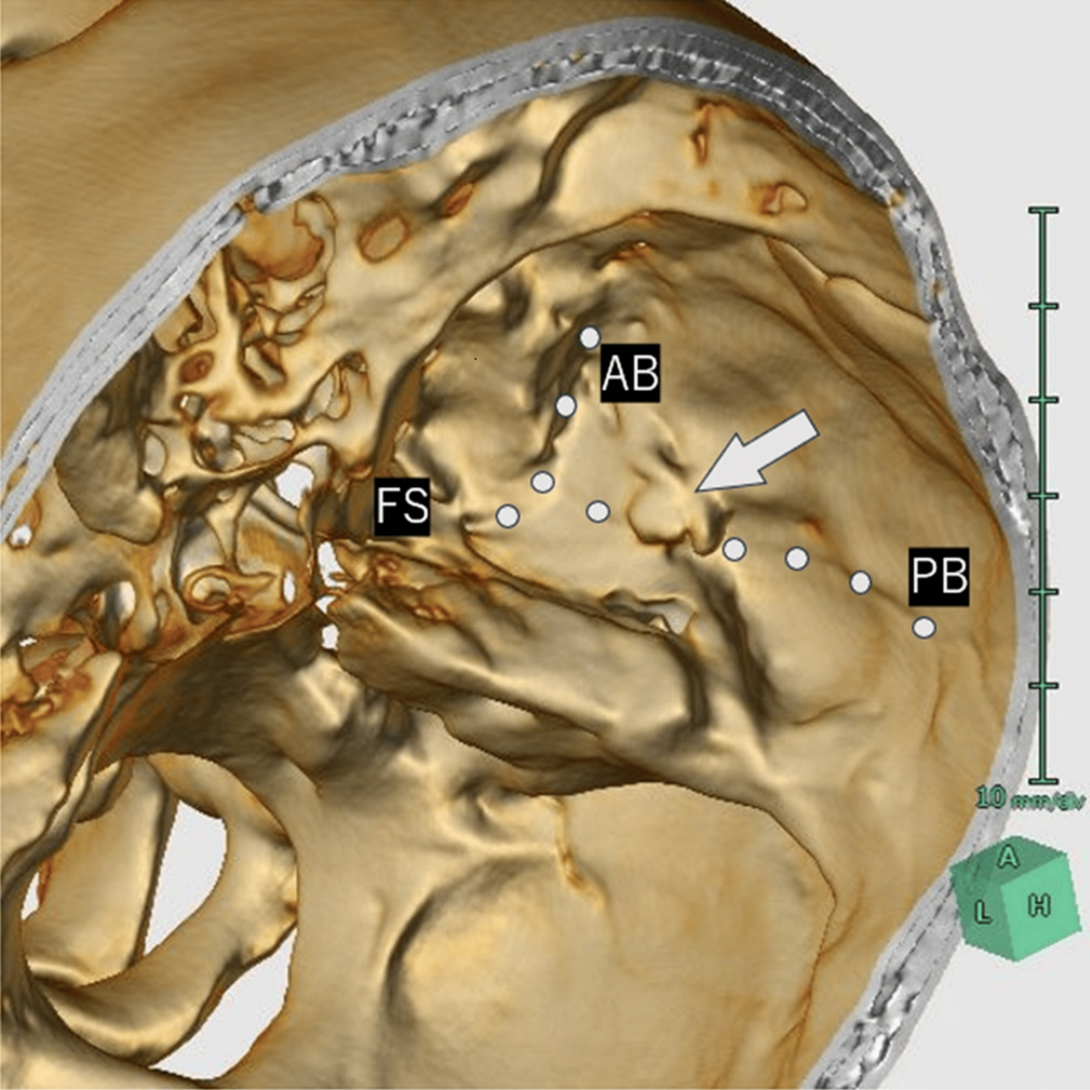
The computed tomography reconstruction image of the head at the time of presentation The gray dotted lines indicate vascular grooves of the middle meningeal artery (MMA). The gray arrow indicates the vascular groove of the posterior branch of the MMA approached the fracture area. In the present case, the length of the main trunk of MMA was approximately 9.3 mm. FS: foramen spinosum, AB: Anterior branch of the MMA, PB: Posterior branch of the MMA.

Postoperatively, coronal and three-dimensional bone images of reconstructive CT revealed fracture lines in the right mandibular fossa and mandibular condyle (Figures 2, 4). A fracture ridge was seen on the middle cranial fossa (the opposite side of the mandibular fossa). The foramen spinosum showed no fracture line, but the vascular groove of the posterior branch of the MMA approached the fracture area (Figures 2, 4). 

The postoperative course was uneventful, with clear consciousness and resolution of motor paralysis the day after surgery. As the mandibular joint fracture was likely to worsen the patient’s occlusal dysfunction, the patient was restricted from opening his mouth. On the 11th postoperative day, the patient could walk independently and was discharged. Neurological abnormalities were not observed. After discharge, oral opening training was started and gradually showed improvement.

## Discussion

Most acute epidural hematomas are thought to be caused by injury to the MMA. However, other causes have been reported, including injury to the middle meningeal vein, venous sinus injury, and bleeding from the diploic vein [2-4]. Some cases involving a skull base fracture at the foramen spinosum due to a lateral head contusion have been reported [2], as well as cases without fractures due to contrecoup injuries [2-4]. In the present case, there was no fracture of the temporal convexity or foramen spinosum, the middle cranial fossa (the opposite side of the mandibular fossa) fracture likely caused by an epidural hematoma by a contusion of the jaw. Although dislocation of the mandibular condyle into the middle cranial fossa by mandibular fossa fracture has been reported, cases of acute epidural hematoma are extremely rare, with only seven cases, including our case [5].

Even when there is a direct impact on the jaw, several safety mechanisms prevent mandibular condyle dislocation into the middle cranial fossa [6]. The temporomandibular joint is originally supported by surrounding muscles and ligaments, making it difficult to penetrate the cranial fossa [6]. Also, where stress is concentrated, fractures of the mandibular head or neck prevent penetration into the cranial fossa [7,8]. However, depending on mechanical and individual factors, the mandibular condyle dislocates into the middle cranial fossa [5,7].

The mechanical factors are as follows. In a closed-mouth condition, the impact is distributed to the maxilla, and the temporomandibular joint alone is not considered to be overloaded. However, in an open mouth condition, a large interferential force is applied to the temporomandibular joint [5,8]. In a jaw median contusion, the mandible is bowed, and the distributed interferential forces cause the bilateral temporomandibular processes to rotate about their sagittal axes [8]. The mechanism is called the “hunting bow concept,” which prevents mandibular condyle dislocation into the middle cranial fossa by fracture of the temporomandibular process [8]. However, contusion on the lateral causes a large impact on the contralateral temporomandibular joint [8].

The individual factors are as follows. The shape of the temporomandibular process changes with age. In youth, the mandibular head is small and round, and the mandibular neck is thick, but as it grows, the mandibular head becomes flat, and the mandibular neck elongates downward and becomes thinner [9]. Also, as the mandible becomes edentulous, bone density in the mandibular head and neck decreases [10]. Therefore, the older the patient is, the more likely a temporomandibular process fracture due to contusion. The risk of temporomandibular joint fossa is higher in younger patients [5,11]. Also, the shape of the fossa plays a role, and cases with a rounded fossa and cases with a well-developed temporal bone pneumatization are more likely to have fossa fractures [5,12]. In addition, also related to mechanical factors, when there is malocclusion, the support of the fossa is unstable, and external forces are transmitted more strongly to the fossa during injury, resulting in dislocation of the mandibular condyle into the middle cranial fossa [5].

In the present case, fractures of the right mandibular fossa and the right mandibular neck were observed due to the contusion of the left side of the jaw. The young male patient had a robust mandible. Despite the stress concentration on the mandibular neck, the neck was not broken but only a crack fracture, which was considered to have resulted in a mandibular fossa fracture.

There have been cases of contralateral mandibular fossa fractures due to the jaw contusion reported in which there was a dislocation of the mandibular condyle into the middle cranial fossa [5-7]. However, only mandibular fossa fractures and dislocation of the mandibular condyle into the middle cranial fossa rarely result in acute epidural hematomas [6]. Even though there has been a case of acute epidural hematoma with mandibular fossa fractures where the patient died after contusion due to a fall [13], most cases with poor prognosis are due to accompanying cerebral contusions from high-energy trauma or due to multiple traumas throughout the body [5,14]. In a review of 116 cases of mandibular head straying into the middle cranial fossa, there were six cases of acute epidural hematoma complications [5]. The etiology underlying the low incidence of acute epidural hematoma compared to the number of mandibular fossa fractures remains unclear. However, it may be related to the variation and individual development of the branches of the MMA.

The MMA branches from the mandibular segment of the maxillary artery and enters the cranium via the foramen spinosum from behind the mandibular process, after which the main trunk branches into anterior and posterior branches [14]. Generally, the bifurcation is considered to occur at about 25 mm from the foramen spinosum. However, there are large racial and individual differences between left and right [15,16]. Ogeng’o et al. examined the bifurcation of the MMA and reported a primary stem measuring 5 mm in 7.2% of cases and less than 10 mm in approximately 30% of cases [17]. This suggests that the primary stem of the MMA is shorter in a minority of cases. In addition, Shotar et al. reported that the anterior branch is more predominant than the posterior branch in many cases [18].

The foramen spinosum is located anteriorly medial to the mandibular fossa. Thus, in the case of a long main trunk, it would not pass over the mandibular fossa but would instead branch into anterior and posterior branches after approaching or reaching the convexity. On the contrary, if the distance from the foramen spinosum to the bifurcation point is short, the posterior branch will run posteriorly outward and directly over the mandibular fossa anatomically and the possibility of damage to the posterior branch of the MMA due to fracture of the mandibular fossa would increase.

However, because the cases of the short primary stem of the MMA are in the minority, mandibular fossa fractures are unlikely to cause an acute epidural hematoma. In the present case, the length of the main trunk was relatively short, approximately 9.3 mm, and retrospective CT showed that the vascular groove of the posterior branch of the MMA reached the convexity after crossing the fracture site (Figure 4).

## Conclusions

We encountered a case of acute epidural hematoma resulting from a mandibular fossa fracture. Acute epidural hematoma caused by mandibular fossa fracture is extremely rare owing to its anatomical location; nonetheless, it may necessitate surgical intervention. Thus, when a mandibular fossa fracture is identified, the complications of acute epidural hematoma should be considered as a possibility, even though it is rare. Although there are few cases of acute epidural hematoma by a mandibular fossa fracture, further studies involving more patients are needed to reveal the etiology of this.
